# Metallurgical Process Simulation and Optimization—2nd Volume

**DOI:** 10.3390/ma18092037

**Published:** 2025-04-29

**Authors:** Jiangshan Zhang, Jiali Tang, Qing Liu

**Affiliations:** State Key Laboratory of Advanced Metallurgy, University of Science and Technology Beijing, Beijing 100083, China; tang_jiali@163.com

As the cornerstone of industrial civilization, metallurgical engineering has consistently driven materials innovation and advanced manufacturing technologies. With the global surge in demand for high-performance steels, lightweight alloys, and sustainable production, traditional metallurgical processes face critical challenges, such as complex process variables, resource shortage, and stringent environmental regulations. This Special Issue, “Metallurgical Process Simulation and Optimization—2nd Volume”, highlights recent advancements in simulating and optimizing metallurgical processes through 19 cutting-edge articles.

Data-driven approaches and reaction kinetics are revolutionizing steelmaking by enhancing parameter optimization, reaction efficiency, and intelligent transformation. Susana et al. [1] identified sulfur partition coefficients as critical indicators for calcium-treated aluminum-killed steel, linking them to slag composition via Pearson correlation analysis. Complementing this, Zhang et al. [2] revealed that 2CaO·SiO₂ layers formed during lime dissolution hinder mass transfer in converter slag, offering strategies to refine slag formulations. Dynamic simulation models further optimized the steelmaking-continuous casting process, reducing total completion time by 8.7 minutes through furnace–machine coordination [3]. In electric arc furnaces, Zhang et al. [4] decoupled nitrogen absorption/desorption kinetics, demonstrating that increasing argon flow rates (100–300 mL/min) quadrupled denitrification constants.

Continuous casting relies on precise flow-field control and solidification uniformity. Li et al. [5] redesigned a four-strand tundish using water modeling, reducing dead zones from 36.47% to 17.59% via optimized baffles. For ultra-wide slab casting, Li et al. [6] correlated casting speed (0.9–1.4 m/min) with surface flow activity, showing a 49% reduction in low-velocity zones. Large eddy simulations quantified mold level fluctuations, establishing a linear relationship between surface velocity (20–60 mm/s) and level oscillations (3–4 mm) [7]. Electromagnetic stirring (M-EMS) and nozzle innovations have been proven to be quite effective. It has been demonstrated that M-EMS mitigates uneven solidification in round billets, while swirl flow nozzles (SFN) reduced slag entrapment by lowering tangential velocity (−15° SFN: 5.07 mm) [8,9]. These advancements highlight the importance of flow-field precision.

Tailoring alloys for corrosion resistance and high-temperature performance remains effective to metallurgy. Liu et al. [10] and Wang et al. [11] enhanced HRB400 rebar and GH4169 superalloys via Cr, RE, and Y additions. Cr stabilized passive films, while RE reduced pitting via (RE)_2_O_2_S inclusions. Yttrium (0.04 *wt*.%) minimized oxidation layer thickness by 30%, improving high-temperature adhesion. In aluminum refining, Aleksandar et al. [12] modeled impurity evaporation (Cd, Hg, Pb, Zn) using logistic functions, identifying transition temperatures (e.g., Zn: 860 °C). Long et al. [13] explored CaO-Al_2_O_3_-BaO-CaF_2_-Li_2_O mold fluxes, showing that Al_2_O_3_ additions reduced devitrification activation energy (314.16→269.46 kJ/mol), accelerating crystallization.

Numerical simulations and sustainable practices are reshaping metallurgy. Tian et al. [14] coupled VOF and DPM models to map bubble dynamics in slab casting, identifying nozzle regions as hotspots for coalescence/fragmentation (average diameter: 0.741 mm). Zhang et al. [15] optimized RH degasser nozzles, achieving 29.8 m^3^/h circulation flow with a 127–87 symmetric layout. Sustainable practices were advanced in this study, where electrolytic reduction in bauxite residue halved activation energy (32.9→17.2 kJ/mol) for Boehmitic leaching, while improving thickening efficiency by 1.5× [16]. Wang et al. [17] linked coke layer thickness to blast furnace permeability, proposing melt droplet metrics to balance gas flow and coke degradation. Wang et al. [18] reviewed induction heating tundishes, emphasizing their role in low-superheat casting for specialty steels—a technology refined since 1984. Wang et al. [19] optimized mold cooling, showing that extending effective length by 100 mm increased heat transfer by 19%, critical for high-speed casting.

This Special Issue underscores the important role of simulation and optimization in addressing metallurgical challenges. From data-driven steelmaking to sustainable resource recovery, these studies bridge fundamental research and industrial application. We believe that these advancements will catalyze further innovations, driving the metallurgical industry toward better efficiency and sustainability.
